# Phylogeny of the Subtribe Monoctonina (Hymenoptera, Braconidae, Aphidiinae)

**DOI:** 10.3390/insects11030160

**Published:** 2020-03-02

**Authors:** Jelisaveta Čkrkić, Andjeljko Petrović, Korana Kocić, Milana Mitrović, Nickolas G. Kavallieratos, Cornelis van Achterberg, Paul D. N. Hebert, Željko Tomanović

**Affiliations:** 1 Institute of Zoology, Faculty of Biology, University of Belgrade, Studentski trg 16, 11000 Belgrade, Serbia; andjeljko@bio.bg.ac.rs (A.P.); korana.kocic@bio.bg.ac.rs (K.K.); ztoman@bio.bg.ac.rs (Ž.T.); 2 Institute for Plant Protection and Environment, Department of Plant Pests, Banatska 33, 11000 Belgrade, Serbia; milanadesancic@yahoo.co.uk; 3 Laboratory of Agricultural Zoology and Entomology, Department of Crop Science, Agricultural University of Athens, 75 Iera Odos str., 11885 Athens, Attica, Greece; nick_kaval@aua.gr; 4 Naturalis Biodiversity Center, 2300 RA Leiden, The Netherlands; c.vanachterberg@xs4all.nl; 5 Centre for Biodiversity Genomics, Biodiversity Institute of Ontario, University of Guelph, 50 Stone Road East, Guelph, ON N1G 2W1, Canada; phebert@uoguelph.ca

**Keywords:** Monoctonina, *Monoctonus*, *Monoctonia*, *Falciconus*, *Harkeria*, barcoding, Aphidiinae, phylogeny

## Abstract

Members of the Monoctonina subtribe have long been neglected in applied studies of the subfamily Aphidiinae, due to their low economic importance, as they do not parasitize pests of cultivated plants. Consequently, data about this group are scarce, including its taxonomy and phylogeny. In the present study, we explore inter- and intraspecific genetic variation of Monoctonina species, including genera *Monoctonus* Haliday 1833, *Monoctonia* Starý 1962, *Falciconus* Mackauer 1959 and *Harkeria* Cameron 1900. We employ two molecular markers, the barcode region of the mitochondrial cytochrome c oxidase subunit I (*COI*) and the D2 region of the 28S nuclear gene (*28S rDNA*), to analyze genetic structuring and phylogeny of all available Monoctonina species, and combine them with morphological data for an integrative approach. We report one new species, and three potentially new species which can be formally described when further specimens are available. Analysis of phylogenetic relationships within the subtribe shows a basal position for the genera *Falciconus* and *Monoctonia*, and the close relatedness of *Harkeria* and *Monoctonus*.

## 1. Introduction

The aphidiine subtribe Monoctonina, comprising genera *Monoctonus* Haliday 1833, *Monoctonia* Starý 1962, *Falciconus* Mackauer 1959, *Harkeria* Cameron 1900, and *Quadrictonus* Starý and Remaudière 1982, has long been omitted from taxonomic and phylogenetic studies. This is probably due to low economic importance of its members, since they do not parasitize pests of cultivated plants, and as a consequence are not commonly used in biological control attempts [1]. Until recently, there was only one revision of European species available [2], while the current identification keys are focused on small geographic regions [1,2,3]. Recently, Čkrkić et al. [1] provided a review of the world Monoctonina based on morphology with descriptions of five new species.

A study based on molecular analysis of the Monoctonina subtribe has not been conducted so far, but some species have been used in molecular studies, mostly those focusing on the entire subfamily Aphidiinae. Most of these studies were focused on relationships between tribes and on determining the basal tribe within the Aphidiinae, rather than relationships within the tribes. A phylogenetic study of Aphidiinae [4] based on elongation factor-1α, cytochrome b and the second expansion segment of the 28S ribosomal subunit supported the grouping of Monoctonina with Trioxina, although only *Falciconus pseudoplatani* (Marshall 1896) was used in the analysis. Smith et al. [5] showed the same relationship between Monoctonina and Trioxina, although with relatively low statistical support, based on the analysis of the mitochondrial NADH1 dehydrogenase gene sequence, with three species from the subtribe used in the analysis (*F. pseudoplatani, Monoctonus paulensis* (Ashmead 1902), and *H. angustivalva* (Starý 1959)). Sanchis et al. [6] used only *Monoctonia vesicarii* Tremblay 1991 in their analysis of Aphidiinae based on *18S rDNA*; a species of the genus *Monoctonus* was also used, but it was later removed from the analysis due to ambiguous results. *Monoctonia vesicarii* was suggested as basal in the clade comprised of Trioxini and Aphidiini. Shi and Chen [7] also used only *M. vesicarii* in their analysis of Aphidiinae based on *16S rRNA*, *18S rDNA*, and *ATPase 6*, and their results suggested the same position of the species as in Sanchis et al. [6]. In a study of Aphidiinae based on the *16S rRNA* gene by Kambhampati et al. [8] five species from the Monoctonina subtribe were examined—*M. vesicarii*, *Monoctonus crepidis* (Haliday 1834), *M. paulensis, F. pseudoplatani*, and *H. angustivalva*. As in previous studies, these species grouped with species of Trioxina and together formed a clade that is basal in the Aphidiini+Trioxini cluster. In a study based on the cytochrome oxidase subunit I gene (*COI*) by Derocles et al. [9], the three Monoctonina species used (*F. pseudoplatani, M. crepidis*, and *M. caricis* (Haliday 1833)) again grouped with Trioxina, but with bootstrap support under 65%. *Falciconus pseudoplatani* appeared closest to *Lipolexis gracilis* Förster 1962; *M. crepidis* grouped with *Binodoxys* Mackauer 1960 species, while *M. caricis* formed a separate branch in the cluster. Rakhshani et al. [10] used the *COI* gene to analyze the genus *Monoctonia*, while *M. crepidis, M. ligustri* van Achterberg 1989, *H. angustivalva*, and *F. pseudoplatani* were also included in the analysis. The analysis showed that genetic distances between species of Monoctonina far exceed what is considered to be a species boundary within the Aphidiinae [9,11]. A new species, *Monoctonia japonica* Rakhshani and Tomanović 2015 was described based on morphological and molecular analyses [10].

The current study aims to further knowledge of the taxonomy and phylogeny of the subtribe Monoctonina, using molecular data from two gene markers (mitochondrial cytochrome c oxidase subunit I (*COI*) and nuclear large subunit *28S rDNA*) combined with morphological measurements of relevant characters. We describe one new species, and report three potentially new species which can be described when more data are available. We also discuss the phylogenetic relationships of genera and species within the subtribe Monoctonina.

## 2. Materials and Methods

### 2.1. Insect Material

Specimens belonging to the subtribe Monoctonina were collected across Holarctic between 1995 and 2017. Samples were collected with Malaise traps in Canada and USA or by collecting parasitized aphids (Serbia, Montenegro, Slovenia, Czech Republic, Belgium, Spain, France, Russia, UK (Wales), USA and Japan) (Supplementary Materials Table S1). Leaves with parasitized aphids were collected from various host plants and kept in plastic boxes covered with nylon mesh for 3–4 weeks or until parasitoid emergence under laboratory conditions (22.5 °C, 16 h L: 8 h D). Emerged parasitoids were immersed in 96% ethanol and kept for later examination. Samples of aphids were kept in 96% alcohol for identification. Parasitoid specimens used in this study are deposited in the collection of Institute of Zoology, Faculty of Biology, University of Belgrade (FBUB) and the Canadian National Collection of Insects, Arachnids, and Nematodes, Ottawa (CNC).

### 2.2. Molecular Analysis

Monoctonina specimens belonging to 22 species were used in the molecular analysis (Table S1). DNA was extracted from 52 individual adult parasitoids using the KAPA Express Extract kit (Kappa Biosystems Inc., Boston, MA, USA) or QIAGEN Dneasy^®^ Blood and Tissue Kit (Qiagen Inc., Valencia, CA, USA) following the manufacturer’s instructions. The barcode region of the *COI* gene was amplified using the universal primers LCO1490 and HCO2198 [12]. In the case of museum specimens, where universal primers were unable to amplify the barcode region due to DNA degradation, specific primer pairs designed to amplify the region as three shorter fragments were used [13]. The D2 region of the nuclear gene for 28S (*28S rDNA*) was amplified from DNA of 33 specimens using the primers 28SD2f [14] and 28SD2r [15].

DNA amplification of *COI* and *28S* was performed in a final volume of 20 μL containing 1 μL of DNA, 11.8 μL of H_2_O, 2 μL of High Yield Reaction Buffer A with 1 x Mg, 1.8 μL of MgCl_2_ (2.25 mM), 1.2 μL of dNTP (0.6 mM), 1 μL of each primer (0.5 μM) and 0.2 μL of KAPATaq DNA polymerase (0.05 U/μL) (Kapa Biosystems Inc., Boston, MA, USA). DNA amplification with specific primers was performed in the same volume, only with 2 μL of DNA. PCR amplification was conducted in an Eppendorf Mastercycler^®^ (Hamburg, Germany) using the following thermal profile for *COI*: initial denaturation at 95 °C for 5 min, followed by 35 cycles of 94 °C for 60 s, 54 °C for 60 s, 72 °C for 90 s (30 s when specific primers were used) and a final extension step at 72 °C for 7 min. The PCR protocol for *28S* was: initial denaturation at 95 °C for 3 min, followed by 32 cycles of 95 °C for 30 s, 48 °C for 30 s, 72 °C for 60 s and a final extension step at 72 °C for 10 min. Purification of PCR products was done either with QIAquick PCR purification Kit (Qiagen Inc., Valencia, CA, USA) or by Macrogen Inc. (Seoul, Korea), while DNA sequencing in both directions was performed by Macrogen Inc. (Seoul, Korea). Additional 132 sequences of *COI* gene were obtained from BOLD database.

Sequences were edited using FinchTV ver. 1.4.0 (www.geospiza.com). Alignment of sequences was conducted using CLUSTAL W algorithm integrated in MEGA 5 software [16]. Sequences of *COI* gene were trimmed to 663 bp, while those of *28S rDNA* were trimmed to 690 bp. All sequences are deposited in GenBank and BOLD (accession numbers and BOLD codes in Table S1).

Calculation of average genetic distances was performed using the Kimura two-parameter method (K2P) of base substitution [17] integrated in MEGA 5 [16].

Phylogenetic relationships among Monoctonina species were assessed using Bayesian inference. MrBayes 3.1.2 software [18] was used to obtain phylogenetic trees based on sequences of *COI* and *28S rDNA* genes, as well as a combined tree using sequences for both genes and coded morphological characters. For all three analyses, the best-fitting model of sequence evolution based on the Akaike Information Criterion was the General Time Reversible model (GTR), as determined by Model Test integrated in MEGA 5. Bayesian inference analysis was conducted by running two Markov Chain Monte Carlo searches, each with one cold and three heated chains. For the tree based on *COI*, the analysis ran for eight million generations, for the *28S* tree for two million generations, and for the combined tree for five million generations. In all cases, sampling was conducted every 1000 generations, while the first 25% of trees were discarded as a burn-in. Convergence of parameters was confirmed by Tracer 1.5.0 program [19], while FigTree 1.3.1 [20] was used to view the consensus tree with posterior probabilities. Haplotype diversity was calculated using DNAsp version 6 [21]. Haplotype networks were constructed using the software Network, version 5.0.0.1 (http://www.fluxus-engineering.com).

The following nine morphological characters or ratios of characters were coded based on original coding strategies or following Tomanović et al. [22,23]:

1. Host range: 0 = Eriosomatinae; 1 = Drepanosiphinae; 2 = Macrosiphini; 3 = Aphidiini
2. Number of antennomeres: 0 = 13; 1 = 14; 2 = 15; 3 = 16; 4 = 17; 5 = 18
3. Flagellomere 1 length/width ratio: 0 = <3; 1 = 3–4; 2 = >4
4. Number of maxillary palpomeres: 0 = 4; 1 = 3
5. Number of labial palpomeres: 0 = 3; 1 = 2; 2 = 1
6. Fore wing pterostigma length/width ratio: 0 = <3; 1 = 3–4; 2 = 4–5; 3 = 5–6; 4 = >6
7. Fore wing pterostigma length/R1 (metacarpus) length ratio: 0 = <3; 1 = 3–4; 2 = >4
8. Fore wing m-cu vein: 0 = fully sclerotized; 1 = partially missing or colorless; 2 = absent
9. Ovipositor sheaths length/width ratio: 0 = <3; 1 = 3–4; 2 = >4

Since the coded morphological characters are based on female specimens, for species where only male specimens were available, the number of antennomeres and ovipositor sheath length/width ratio were coded as missing data.

## 3. Results

### 3.1. Molecular Analysis

Analysis of *COI* sequences obtained from 183 specimens of Monoctonina revealed 267 variable sites, with 243 of those parsimony informative, while 24 were singleton positions. Among these specimens, 172 were identified to a species, based on morphological and molecular data. Eleven sequences that were initially identified to genus level represent four distinct taxonomic units, based on genetic differences between them and other species in the analysis. Based on the *COI* sequences, one new species is described below, and three potentially new species are reported (Appendix A).

**Figure 1:**
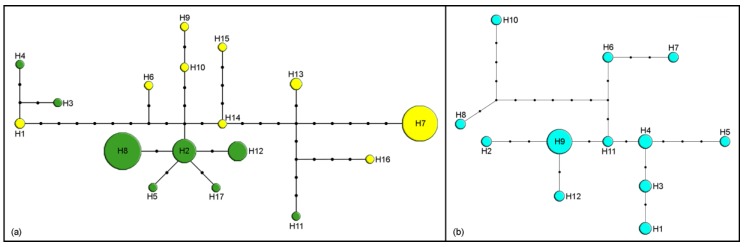
Haplotype networks based on cytochrome c oxidase subunit I (*COI*) sequences. Size of circles indicates the number of specimens with a haplotype; black dots represent one nucleotide substitution: (**a**) *M. washingtonensis* (yellow—west haplotypes, green—east haplotypes); (**b**) *M. paulensis*.

Seven of 22 species were represented by just one individual, but more than one haplotype was present in all 15 species represented by two or more specimens. The highest number of haplotypes was detected in *M. washingtonensis* Pike and Starý 1995 (17) in 74 analyzed specimens. Over 50% of analyzed mitochondrial barcode sequences belong to haplotypes H7 and H8 (52.6%, Figure 1a). In 25 sequences of *M. paulensis*, 12 haplotypes were detected (Figure 1b).

**Figure 2:**
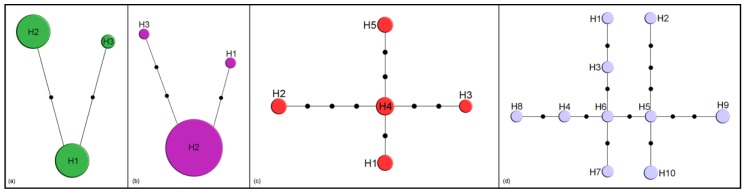
Haplotype networks based on cytochrome c oxidase subunit I (*COI*) sequences. Size of circles indicates the number of specimens with a haplotype; black dots represent one nucleotide substitution: (**a**) *M. crepidis*; (**b**) *M. caricis*; (**c**) *M. brachyradius*; (**d**) *F. pseudoplatani*.

**Figure 3:**
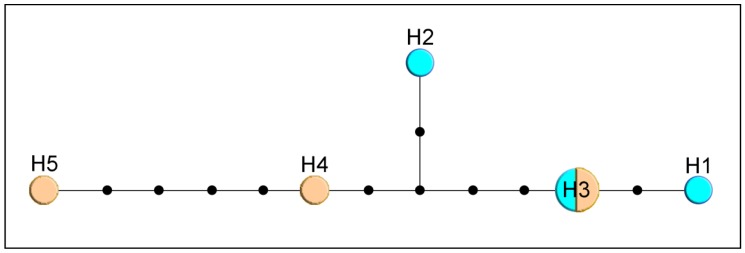
Haplotype network based on *COI* sequences of specimens identified as *M. ligustri* (blue) and *M. mali* (beige). Size of circles indicate the number of specimens with a haplotype; black dots represent one nucleotide substitution.

By comparison, only three haplotypes were detected in *M. crepidis* (17 specimens, Figure 2a) and *M. caricis* (47 specimens, Figure 2b). Five haplotypes were detected among 12 sequences from *M. brachyradius* Čkrkić, Petrović and Tomanović 2019 (Figure 2c), while ten haplotypes were detected among 12 specimens of *F. pseudoplatani* (Figure 2d). Specimens initially identified as *M. ligustri* and *M. mali* van Achterberg 1989 share five haplotypes (8 analyzed sequences, Figure 3).

**Figure 4:**
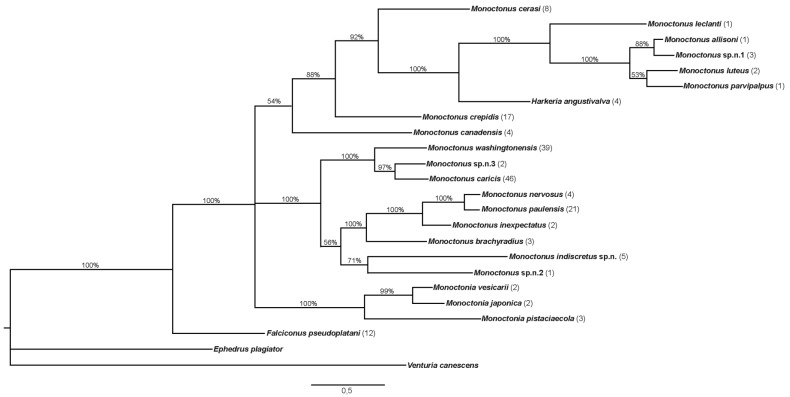
Phylogenetic tree constructed based on sequences of *COI*. The number of specimens of each species is shown in parentheses. Bayesian posterior probabilities are shown above branches.

Phylogenetic tree constructed in MrBayes based on sequences of the *COI* gene is shown in Figure 4. Number of specimens used for every species is shown in parentheses.

The topology of the tree shows two main groups of *Monoctonus* species, joined by *H. angustivalva*, while *F. pseudoplatani* and *Monoctonia* species form separate branches on the tree. *Monoctonus leclanti* Tomanović and Starý 2002, *M. allisoni* Pike and Starý 2003, *M. luteus* Čkrkić, Petrović and Tomanović 2019, *M. parvipalpus* Čkrkić, Petrović and Tomanović 2019, and *Monoctonus* sp. n. 1 cluster closely and are joined by *H. angustivalva* as a sister species, while *M. ligustri* and *M. mali* (shown in the tree as *M. cerasi* (Marshall 1896)), *M. canadensis* Čkrkić, Petrović and Tomanović 2019 and *M. crepidis* are on separate branches to form one *Monoctonus* clade. In the second clade, *M. washingtonensis*, *M. caricis*, and *Monoctonus* sp. n. 3 cluster together, as do *M. nervosus* (Haliday 1833), *M. paulensis*, *M. brachyradius*, and *M. inexpectatus* Čkrkić, Petrović and Tomanović 2019. *Monoctonus indiscretus*, sp. n. and *Monoctonus* sp. n. 2 form a separate branch in this clade.

Table 1 shows interspecific genetic distances for *COI*, while distances between genera are shown in Table 2. With the exception of low distances between *M. nervosus* and *M. paulensis* (2.7%), *M. allisoni* and *Monoctonus* sp. n. 1 (3.5%), and specimens initially identified as *M. ligustri* and *M. mali* (0.4%), interspecific distances range from 4.5% between *Monoctonia vesicarii* and *M. japonica*, to 23.2% between *Monoctonus parvipalpus* and *M. japonica*.

**Figure 5:**
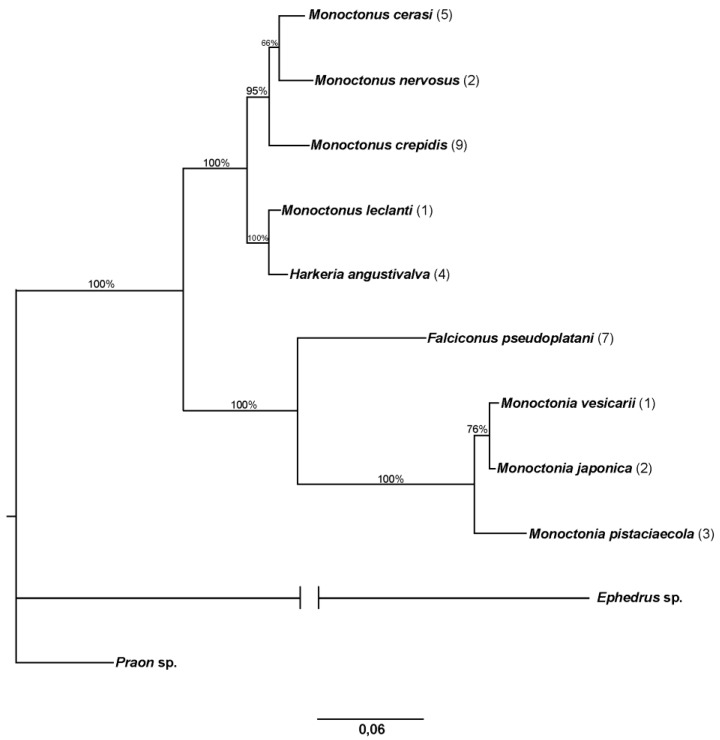
Phylogenetic tree based on *28S rDNA*. The number of specimens of each species is shown in parentheses. Bayesian posterior probabilities are shown above branches.

Sequences for *28S rDNA* were obtained from 33 specimens and aligned to 690 bp. Figure 5 shows the phylogenetic tree constructed in MrBayes. As expected for a tree based on this gene, more species are clustered. Specimens identified as *M. ligustri* and *M. mali* (=*M. cerasi*) have identical sequences. *Monoctonus crepidis, M. nervosus*, and *M. cerasi* all group together, with *M. leclanti* and *H. angustivalva* joining as a sister clade. *Falciconus pseudoplatani* and *Monoctonia* species make up a separate clade on the tree.

Genetic distances for *28S rDNA* between genera are similar to those for *COI*, with the exception between *Monoctonus* and *Harkeria* (4.3%) (Table 2). Distances calculated based on the same gene within *Monoctonus* are much lower than those for *COI*, ranging from 0% between specimens initially identified as *M. ligustri* and *M. mali*, to 4.9% between *M. leclanti* and *M. paulensis* (Table 3).

**Figure 6:**
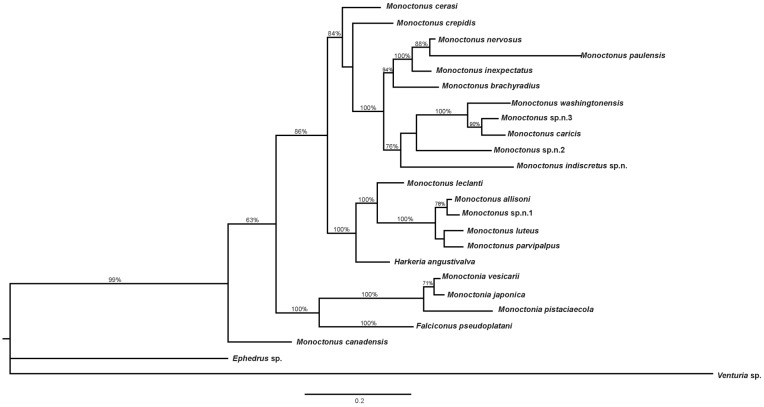
Phylogenetic tree based on *COI*, *28S rDNA* and coded morphological characters. Bayesian posterior probabilities are shown above branches.

Combined phylogenetic tree based on *COI* and *28S* DNA sequences and morphological characters constructed in MrBayes is shown in Figure 6. *Falciconus* and *Monoctonia* are separated from the clade comprised of *Monoctonus* and *Harkeria. Monoctonus crepidis*, *M. cerasi*, and *M. canadensis* form separate branches in this clade, while other species form two groups. One group consists of *M. allisoni, M. luteus, M. parvipalpus*, and *Monoctonus* sp. n. 1 grouped together, with *M. leclanti* and *H. angustivalva* as sister species. The second group is comprised of two clades, one containing *M. nervosus, M. paulensis, M. brachyradius*, and *M. inexpectatus*, and the other one containing *M. washingtonensis, M. caricis, M. indiscretus* sp. n., *Monoctonus* sp. n. 2, and *Monoctonus* sp. n. 3.

### 3.2. Description of a New Species

**Figure 7:**
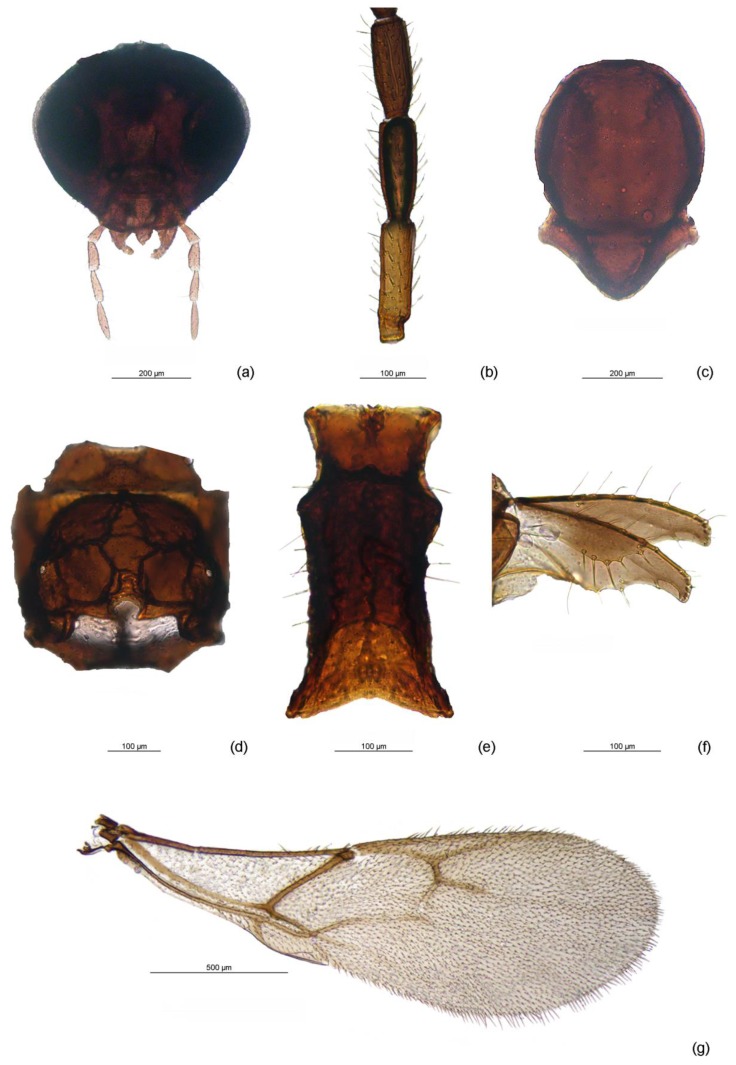
*Monoctonus indiscretus* sp. n., female: (**a**) head; (**b**) flagellomeres 1–3 (F1–F3); (**c**) mesoscutum; (**d**) propodeum; (**e**) petiole; (**f**) ovipositor sheath; (**g**) fore wing.

*Monoctonus indiscretus* Čkrkić, Petrović and Tomanović, sp. n. Figure 7.

*Diagnosis*. This species is morphologically very similar to *M. nervosus* and *M. paulensis*. It cannot be easily distinguished from these two species, as most character states overlap. The analyzed female specimens of the new species have a yellow F1. In contrast, *M. paulensis* has only a yellow narrow basal ring of F1 and the remainder brown, and *M. nervosus* usually has a yellow F1 and basal third of F2. Ratio of length and width of F1 is 3.0–3.2 in *M. indiscretus*, while it ranges between 3.4 and 4.3 in *M. nervosus* and 3.6–4.7 in *M. paulensis*. Pterostigma length/R1 length is 2.4 in *M. indiscretus*, 1.8–2.3 in *M. nervosus* and approx. 2.5 in *M. paulensis*, although the range of variation in *M. paulensis* (2.1–3) overlaps with *M. indiscretus* measurements.

#### 3.2.1. Female

*Head* (Figure 7a). Eyes oval, medium sized, sparsely setose. Malar space equal to 0.23 of longitudinal eye diameter. Tentorial index approx. 0.2. Clypeus oval with 10–11 setae. Maxillary palps with four palpomeres, labial palps with three palpomeres. Antenna with 16 antennomeres, filiform, setae on flagellomeres semi-erect, subequal to half of segment diameter. Flagellomere 1 (F1) 3.0–3.2 times as long as wide, with 0–2 longitudinal placodes. F2 approx. 2.5 times as long as wide, without longitudinal placodes. F3, F4, and F5 with 4–5, 3–4, and 3–5 longitudinal placodes, respectively. F1 length equal to F2 length (Figure 7b).

*Mesosoma*. Mesoscutum without notaulices (Figure 7c), dorsal surface scarcely setose. Head width/mesoscutum width ratio 1.3. Propodeum areolated, with narrow central pentagonal areola (Figure 7d).

*Fore wing*. (Figure 7g). Wing length approx. 2 mm, width approx. 0.7 mm. Pterostigma narrow, 6.8 times as long as wide and 2.4 times as long as distal abscissa of R1. Vein m-cu visible, vein 2RS visible, colorless in second half. Veins r and 3RS distinct.

*Metasoma*. Petiole (= first tergite) 2 times as long as wide at spiracles (Figure 7e). Dorsal disc of petiole moderately rugose, especially in middle third, and with 7–8 long setae on the sides. Ovipositor sheath distinctly plough-share shaped (Figure 7f). Ovipositor sheath length/width ratio 2.7.

*Color*. Head brown, eyes black. Scapus and pedicel brown, F1 yellow, remainder of antenna brown. Mesonotum, propodeum, and legs brown. Wings hyaline with brown venation. Petiole and rest of metasoma brown, ovipositor sheaths light brown. Body length 2.6 mm.

#### 3.2.2. Male

*Head*. Eyes oval, medium sized, sparsely setose. Malar space equal to 0.3 of longitudinal eye diameter. Tentorial index approx. 0.4. Clypeus oval with 9–10 setae. Maxillary palps with four palpomeres, labial palps with three palpomeres. Antenna damaged, flagellomeres 1–5 present; filiform, setae on flagellomeres semi-erect, subequal to segment diameter. Flagellomere 1 (F1) 2.6 times as long as wide, with 4 longitudinal placodes. F2 2.4 times as long as wide, with 4 longitudinal placodes. F3, F4, and F5 with 3–5, 3 and 4 longitudinal placodes, respectively. F1 length equal to F2.

*Mesosoma*. Mesoscutum without notaulices, scarcely setose. Head width/mesoscutum width ratio approx. 1. Propodeum areolated, with central pentagonal areola.

*Fore wing*. Wing length approx. 1.4 mm, width approx. 0.5 mm. Pterostigma narrow, approx. 6.2 times as long as wide and 2.6 times as long as distal abscissa of R1. Vein m-cu visible in first third, the rest visible but colorless. 2RS visible in first half. Veins r and 3RS distinct.

*Metasoma*. Petiole approx. 2.1 times as long as wide at spiracles. Dorsal disc of petiole moderately rugose, with 2–3 long setae on the sides.

*Color*. Head and antenna brown, mouthparts light brown. Mesonotum, propodeum and legs brown. Wings hyaline with brown venation. Petiole and rest of metasoma light brown. Body length 2.1 mm.

##### Host: Unknown

*Distribution*: Eastern Canada, United Kingdom, Norway

*Etymology*: The name of the new species, *indiscretus*, comes from latin for *indistinguishable*, since it cannot easily be differentiated morphologically from related species *M. nervosus* and *M. paulensis*.

*Holotype*. Female. Canada, Prince Edward Island, Prince Edward Island National park, Woodland Trail/Long Point, 15 V 2013, coll. P. Ayles, Malaise trap. Holotype slide mounted and deposited in the Canadian National Collection of Insects, Arachnids and Nematodes, Ottawa (CNC). *Paratypes*: 1 female, Canada, Ontario, Cloyne, North Addington Education Centre, 8 V 2015, coll. Melissa Randle, Malaise trap; 1 male, Canada, Ontario, Mississauga, Lorne Park Public School, 2 X 2015, coll. P. Kossowski, Malaise trap. Paratypes slide mounted and deposited in the collection of Institute of Zoology, Faculty of Biology, University of Belgrade (FBUB).

Descriptions of three potentially new species of Monoctonina are given in Appendix A.

## 4. Discussion

Species in the subtribe Monoctonina have generally been neglected in phylogenetic and taxonomic studies, due to their low economic importance and lack of use as biological control agents, as well as the mostly high montane distribution of their populations [1]. Phylogenetic studies of the subfamily Aphidiinae generally examined only a few Monoctonina species, or entirely excluded them from the analysis [4,5,6,7,8]. In this study, we used sequences of the barcode region of the mitochondrial cytochrome c oxidase subunit I, a gene commonly employed in phylogenetic and taxonomic studies of the Aphidiinae wasps [9,10,11,24,25,26,27]. The second marker used in this study, the nuclear large subunit 28S rDNA, has been used in studies examining phylogenetic relationships within the Braconidae [4,26,28,29,30,31]. This gene is useful for inferring phylogenies above the species level, since it has a lower rate of evolution than the mitochondrial genes used for species delimitation. It can, in fact, be used to analyze evolutionary events in Paleozoic and Mesozoic, while some of its faster-evolving regions can reconstruct more recent events [32]. Analysis of the two molecular markers, combined with morphological data, helped to clarify relationships among genera and species of the subtribe Monoctonina. The genera *Falciconus* and *Monoctonia* are separated from the rest of the subtribe, while *Monoctonus* and *Harkeria* are closely related. Average genetic distances for both analyzed genes are high, implying that the subtribe is phylogenetically old (Table 1, Table 2 and Table 3).

There have been numerous studies showing a relationship between genetic distances based on mitochondrial genes and species ages. The most often cited study [33] suggests 2.3% divergence per 1 million years. However, this is often cited uncritically in studies employing only the *COI* gene, while the mentioned study only examined a partial sequence of *COI*, and the majority of the data was for cytochrome c oxidase subunit II [34,35]. Studies using only the *COI* gene show different results, ranging from <1% to >3% per 1 my, depending on the methods used for molecular clock calibration [35]. Calibrating based on habitat and analyzing *Tetraopes* Dalman (Coleoptera, Cerambycidae), Farell [36] suggested a 1.5% divergence per 1 my, and similar results were obtained by Quek et al. [34], analyzing codiversification between *Crematogaster* Lund ants and *Macaranga* sp. The study by Machado et al. [37], analyzing phylogenetic relationships and biogeography of Agaonidae wasps (Hymenoptera, Chalcidoidea), reported a substitution rate of 1.9% per 1 my. By comparison, Papadopoulou et al. [35] reported 3.5% divergence per 1 my, based on biogeographical calibrations for island populations of Tenebrionidae. As with the study by Brower [33], these data should be compared with caution. Although the same gene was examined, the studies targeted different groups of organisms, habitats, and employed different calibration methods and substitution models, making it difficult to reach general conclusions. Furthermore, analyses employing a molecular clock approach, in the absence of fossil data and reliably determined time distances, often include a set of assumptions that can greatly influence the results and their interpretation [38,39,40,41]. However, if we compare *COI* distances obtained here with results from previous studies, it is probable that the separation of Monoctonina genera occurred sometime in Miocene, which is concordant with the fossil finding of *Promonoctonia quievreuxi* (Quillis 1940) from the Oligocene.

Genetic distances based on the *28S rDNA* between *Falciconus* and the other genera are very high, comparable with those based on *COI* (Table 2). Based on the COI gene, *F. pseudoplatani* differs from almost all other species by over 15% (Table 1). Molecular results combined with morphological data, especially the ovipositor shape which differs from all other Monoctonina species [1], indicate that *Falciconus* is a separate clade of Monoctonina. In all phylogenetic trees, *F. pseudoplatani* has a basal position. Another important characteristic of this species that separates it from the rest of the tribe is its host range. While other members of Monoctonina parasitize aphids from the subfamilies Aphidinae (tribes Aphidini and Macrosiphini) and Eriosomatinae, *F. pseudoplatani* is a specialized parasitoid of Drepanosiphinae aphids on *Acer* spp. Haplotype analysis of *F. pseudoplatani* revealed high diversity with 10 haplotypes among 12 analyzed sequences (Figure 2d), separated geographically. Haplotypes H2, H5, H6 and H7 are recorded in Serbia, H3 and H4 in Czech Republic, H1 in Montenegro, H8 in Germany, while H9 and H10 are known from France. Since all specimens were reared from the same hosts, *Drepanosiphum* spp., it is evident that geographical factors play a role in the diversification of *F. pseudoplatani* populations. There is a growing number of studies showing a similar situation, highlighting the importance of inclusion of geographical aspects in population diversity studies [24,42,43,44].

The genus *Monoctonia* includes three species, all specialized parasitoids of gall forming aphids. A comprehensive revision of the genus is given by Rakhshani et al. [10]. The molecular data used in this study show that *Monoctonia* has a basal position within the subtribe (Figure 4, Figure 5 and Figure 6). This is also supported by some of the plesiomorphic morphological characters, such as a subquadrate petiole, triangular pterostigma and short and thick flagellomeres [10]. Furthermore, the aphid hosts parasitized by *Monoctonia* species are considered basal within Aphididae [45,46].

Most morphological characters of *H. angustivalva* are shared with *Monoctonus*, except for the ovipositor shape, which is more similar to *Falciconus* [1,3]. Another character used for the distinction of *Harkeria* from *Monoctonus* is the absence of the propodeal areola in *Harkeria*, although *M. hispanicus*, known only from type material, shares this character state with *H. angustivalva* [47]. The *28S rDNA* results indicate a close relatedness between *H. angustivalva* and species of *Monoctonus*, with distances similar to interspecific distances within *Monoctonus* (Table 2 and Table 3). Based on *COI*, *H. angustivalva* is closest to the group containing *M. leclanti, M. allisoni, M. parvipalpus, M. luteus*, and *M. indiscretus* (Table 1, Figure 4). *Monoctonus leclanti* was the first species of Monoctonina with a narrower ovipositor sheath to be described, and as such was considered transitional between the two genera [3,48]. Other species (*M. allisoni, M. luteus*) have an even narrower ovipositor sheath [1]. Although still high, genetic distances for *COI* between *H. angustivalva* and *Monoctonus* species are not significantly higher than *Monoctonus* interspecific distances. Based on morphological and molecular analyses, as well as aphid hosts that are shared with *M. crepidis* and *M. hispanicus* (*Nasonovia* spp.), *H. angustivalva* should probably be placed within *Monoctonus*, in the group of species with narrow ovipositor sheaths. It should be noted that *Harkeria* contains another species, *H. rufa* Cameron 1900, known from the UK (holotype), Finland, and USA. Unfortunately, specimens of *H. rufa* were not available for analysis.

The genus *Monoctonus* includes most species of Monoctonina. Genetic distances based on the *COI* gene are very high (Table 1). Until recently, an average distance higher than 2–3% was considered to be enough for the separation of species within the Aphidiinae [11,25]. Recent studies show that a high genetic distance based on *COI* in Aphidiinae is not uncommon [24,27], and that the divergence rate probably depends on the age of the analyzed group.

The recently described *M. canadensis* is positioned at the base of the combined phylogenetic tree (Figure 6), although with statistical support of 63%. Interestingly, this species has the lowest distance based on *COI* with *F. pseudoplatani* (13%, Table 1). However, morphological characters undoubtedly place it in the genus *Monoctonus*, specifically in the *nervosus* group s.l. [1]. Further research is needed to determine the host range of this species, to better understand its position within the subtribe.

*Monoctonus* species form two large clades on phylogenetic trees, based on *COI* and on combined morphological and molecular data (Figure 4 and Figure 6). One of the two clades comprises *M. washingtonensis, M. caricis, M. nervosus, M. paulensis, M. inexpectatus, M. brachyradius, M. indiscretus* sp. n., *Monoctonus* sp. n. 2 and *Monoctonus* sp. n. 3. *Monoctonus* sp. n. 2 differs from all other species of the subtribe, in having maxillary palps with three palpomeres. Its fore wing venation is reduced, and the remaining veins (r and RS) are similar to genera *Binodoxys* and *Trioxys* Haliday 1833. However, the shape of its fore wing pterostigma is typical for *Monoctonus*. Shape of petiole is also different from other Monoctonina species; while all other Monoctonina have a petiole length/width ratio at least 2 [1], this species has a distinctly shorter and wider petiole, with very prominent spiracular tubercles. Only male specimens of this potentially new species were found, and without host data, so it is impossible to reach solid conclusions based on morphological characters. Because females of Aphidiinae tend to have a more slender body, it is possible that the petiole shape of a female of *Monoctonus* sp. n. 2 is closer to that typical for other Monoctonina species. Further sampling efforts are necessary to confirm this hypothesis. Based on *COI*, an average distance of 15.7% (Table 1) clearly separates this taxon from other species in the subtribe.

*Monoctonus nervosus* group s.s. comprises *M. nervosus* and *M. paulensis*, morphologically similar species which are separated geographically and ecologically [1]. Analyzed specimens of *M. paulensis* show high intraspecific diversity at *COI*, with 12 haplotypes among 25 individuals (Figure 1b). Host data are not available for all analyzed specimens. However, for those specimens with known aphid hosts, there is no connection between the hosts and the haplotype distribution. Haplotype distribution also does not show a connection with geographical distribution, since both haplotypes with more than one specimen were detected in different populations. More specimens from different populations and aphid host data are needed to infer the causes of genetic structuring of populations of *M. paulensis*.

This study revealed a new species belonging to this group, *M. indiscretus* sp. n. (Figure 7). This species cannot be easily differentiated from *M. nervosus* and *M. paulensis* based on morphology, but it is clearly separated from by the barcode gene—13.1% divergence from *M. nervosus* and 13.7% from *M. paulensis* (Table 1). One morphological difference among the three species, the color pattern of F1 (yellow ring at the base, whole F1 yellow or F1 and part of F2 yellow), has been used in delineation of Aphidiinae species and is considered a relatively stable character [49,50]. The geographical distribution of the new species further complicates the situation within this group—while *M. nervosus* is only known from the Palaearctic and *M. paulensis* from the Nearctic, sequences of *M. indiscretus* sp. n. were recovered from specimens collected in Canada, United Kingdom and Norway. Even though it cannot be easily distinguished morphologically, the high genetic distance from known taxa warrants its recognition as a new species. With the development of DNA sequencing technologies in recent years, gene sequences are becoming an important tool in taxonomic studies. An increasing number of studies have shown the importance of including DNA barcode sequences in species descriptions, whether as the sole diagnostic character when morphological differences are not apparent, or as an additional character coupled with morphological analysis, highlighting the importance of an integrative approach in taxonomy [51,52,53]. Cross-breeding experiments could give a definite answer about the status of those three related species.

*Monoctonus brachyradius* and *M. inexpectatus* are members of the *nervosus* group s.l., differing from *M. nervosus* by a shorter R1 vein, and in the case of *M. inexpectatus*, by labial palps with two palpomeres [1]. Both group with *M. nervosus* and its closely related species in the constructed phylogenetic trees, and show slightly lower genetic distances based on *COI*—*M. inexpecatus* differs from *M. nervosus* by 7% (6.5% from *M. paulensis*), while in the case of *M. brachyradius* the distance is 9.4% (9.5%) (Table 1). The two species have so far only been recorded from North America, as *M. paulensis*. Since Monoctonina is considered an old group within Aphidiinae, it is probable that these North American species separated early in the evolution of the genus and had enough time to accumulate current levels of interspecific differences. All *M. brachyradius* specimens were collected in eastern Canada, with nine of ten specimens from Quarry Island (ON), a boreal forest habitat. Among these specimens, five *COI* haplotypes were recovered (Figure 2c), making it likely that further diversity would be detected with more specimens. The habitat in which the specimens were found matches the general habitat requirements for Monoctonina, which are usually found in forest habitats and on high mountains [1].

Another clade on phylogenetic trees includes *M. caricis, M. washingtonensis*, and *Monoctonus* sp. n. 3, species whose labial palps have two palpomeres. *Monoctonus caricis* and *M. washingtonensis* also share aphid hosts, both parasitizing aphids common on grasses and cereals. For example, both species are parasitoids of *Rhopalosiphum padi*; *M. caricis* has been found on *Hyalopteroides humilis* (Walker) and *Sitobion fragariae* [2], while *M. washingtonensis* has been recorded from *Diuraphis noxia* (Kurdjumov) [54]. The aphid host and female specimens of *Monoctonus* sp. n. 3 are unknown. Genetic distances based on *COI* between the three species are lower than average for the subtribe (Table 1). *Monoctonus caricis* occurs in the western Holarctic [2,55], while the other two species are so far only known from North America [54]. Starý [55,56] noted that *M. caricis* probably has a European origin, and that it likely colonized to North America from Iceland, where it is often recorded. Haplotype diversity within *M. caricis* is very low, as it is represented by only three haplotypes (Figure 2b). Because 45 of the 47 specimens collected in Canada, France, Germany and Norway share the same haplotype (H2), North American populations of *M. caricis* may well represent a recent human-mediated range extension of a species formerly restricted to Europe. The other two specimens belong to haplotypes H1 and H3, and were collected in Germany and Canada, respectively. It seems unlikely that *M. washingtonensis* and *Monoctonus* sp. n. 3 occur in Europe as there have been numerous studies of Aphidiinae associated with cereals in Europe [50,57,58,59,60,61]. It is more difficult to rule out the possibility that all three species have a North American origin. *Monoctonus washingtonensis* exhibits very high intraspecific diversity, with 17 haplotypes among 74 sequences (Figure 1a). Two haplotypes comprise >50% of the total (H7—19 specimens, H8—20 specimens), while others are represented with fewer specimens (H2—11 specimens, H12—8, H13—3, H1—2, remaining haplotypes—1). The two dominant haplotypes are geographically separated—H7 was encountered in several populations in western Canada, while H8 was found in one population in eastern Canada. Haplotypes H2, H3, H4, H5, H11, H12, and H17 were found in eastern populations, while H1, H6, H9, H10, H13, H14, H15, and H16 were only recorded in the west of Canada. The structuring of haplotypes shows a clear geographic differentiation, and it is probable that all haplotypes are derived from the two most frequent ones, H7 and H8.

The second large clade comprises the remaining *Monoctonus* species (Figure 4 and Figure 6) and *H. angustivalva*. *Monoctonus allisoni* and *Monoctonus* sp. n. 1 have a low genetic distance based on the *COI* gene (3.5%), compared to much higher average distance for the whole subtribe (Table 1). Both have only been recorded in North America [62], so a Nearctic origin can be assumed. However, female specimens of *Monoctonus* sp. n. 1 and host data should be included in the analysis for more concrete conclusions. *Monoctonus luteus* and *M. parvipalpus* are also reported only from the Nearctic, again without host data [1], and exhibit somewhat lower genetic distances between themselves and *M. allisoni* (Table 1). Species from this group, *M. luteus, M. parvipalpus, M. allisoni* and *M. leclanti* as a sister species, possess a more narrow ovipositor sheath. The first one to be described, *M. leclanti*, has already been proposed as a transition species between *Harkeria* and *Monoctonus* [48], and the discovery of new species with this character state supports this conclusion.

Two *Monoctonus* species form separate branches on the phylogenetic trees, and are positioned as sister species to different groups, depending on the data employed for tree construction. *Monoctonus crepidis* and *M. cerasi* join the group with narrower ovipositor sheaths in the tree based on *COI* sequences (Figure 4), while they group with the *nervosus* group in the tree based on molecular and morphological data (Figure 6). *Monoctonus crepidis* is a relatively common parasitoid of *Nasonovia* spp. aphids on *Hieracium* spp. and related plants in deciduous forests. It often occurs in mixed populations with *H. angustivalva*, where it is usually dominant [63]. Although it is abundant, this species exhibits very low haplotype diversity, with only three haplotypes recorded in this study (Figure 2a). It appears this species represents a separate evolutionary line within the subtribe, based on its very high genetic distance from other members (Table 1) and certain morphological characters, such as 13-segmented antennae and a distinctly wide pentagonal areola on the propodeum [1].

*Monoctonus ligustri* and *M. mali* were described from specimens previously identified as *M. cerasi* [2]. A recent study [1] showed that the two species are morphologically indistinguishable and that the examined specimens morphologically correspond to *M. cerasi*. In this study, molecular analyses of the two species showed identical sequences for both molecular markers and haplotype distribution independent of aphid hosts (Figure 3). Based on these results, *M. ligustri* and *M. mali* are designated as junior synonyms of *M. cerasi*.

Genetic distances for *COI* between members of the Monoctonina subtribe are very high (Table 1), much higher than what is considered enough for the separation of species [11,25], suggesting that the group is an old one within the subfamily Aphidiinae. While evolutionary younger genera, such as *Aphidius* Nees 1818 and *Lysiphlebus* Förster 1862, show considerably lower interspecific distances at *COI* [11,25,26], recent studies show high interspecific distances within older genera, such as *Ephedrus* Haliday 1833 [27], and within groups closely related to the Monoctonina, such as the genera *Binodoxys* and *Trioxys* [24]. These results show that species cannot be delimited based on a fixed value determined for a subfamily. Instead, when analyzing potentially different species, it is best to use an integrative approach, including evolutionary, biological and ecological specificities of analyzed genera, subtribes, or tribes.

This study has revealed some cases where morphological and molecular analyses provide differing perspectives, a situation that is commonly encountered in the Aphidiinae [26,64,65,66]. Prior research on the Aphidiinae wasps has revealed some cases of discordance between patterns of diversification in the barcode region and morphological/ecological traits. For example, molecular data does not support separation of different phenotypes into species in the *fabarum* group of *Lysiphlebus*, which has sexual and asexual populations, even though those phenotypes have clear morphological and ecological differences [26,66,67]. Moreover, the *COI* distances between *L. testaceipes* (Cresson 1880) and *L. fritzmuelleri* Mackauer 1960 are similar to intraspecific distances for *L. testaceipes*, even though these two species have differing morphology, ecology and geographical origins [26]. Tomanović et al. [65] did not find a clear correlation between morphological and molecular data when they analyzed fore wing shape and the *COI* gene in three closely related species of *Aphidius*. Work on the *Aphidius urticae* s.s. group revealed its separation into three distinct phylogenetic lines (*A. urticae* Haliday 1834, *A. rubi* Starý 1962 and *A. silvaticus* Starý 1962), but morphological differences were not apparent [68]. Another example of molecular data not being informative involved *A. microlophii* Pennacchio and Tremblay 1987 and *A. ervi* Haliday 1834. Although morphologically very similar, these two species use different aphid hosts. *Aphidius microlophii* has only been reported from two aphid hosts, *Microlophium carnosum* (Buckton) and *Wahlgreniella ossiannilssoni* Hille Ris Lambers [69,70], while *A. ervi* parasitizes numerous aphids, but not the hosts of *A. microlophii*. While it has been established that the two species are separate [71], their *COI* sequences are identical [9]. These differences between morphological and molecular results in Aphidiinae merit further investigation. It is possible that the analysis employing only two molecular markers may not be sensitive enough to detect differences in traits that are probably determined by a bigger number of genes, such as fore wing shape [72,73]. Within Monoctonina, exceptions can be found in groups consisting of *M. nervosus, M. paulensis, M. inexpectatus*, and *M. brachyradius* on one hand, and *M. allisoni, M. luteus, M. parvipalpus, M. leclanti*, and *H. angustivalva* on the other. These groups show general concordance between morphological and molecular results. However, the overlap between molecular and morphological results suggests that it would be best to employ additional molecular markers and host data for these species to reach more definite conclusions. Future molecular work concerning Aphidiinae should focus on developing a multi-locus genotyping approach by targeting the most informative genes and regions, to gain a clearer insight into evolutionary history and phylogenetic relationships.

## 5. Conclusions

The subtribe Monoctonina represents an old group within the subfamily Aphidiinae, based on both the deep genetic distances among its members and some morphological characters. Genetic distances between Monoctonina species are higher than what was, until recently, considered a species boundary within Aphidiinae. The genera *Falciconus* and *Monoctonia* are basal within the subtribe, showing greater genetic distances than average for the subtribe, some plesiomorphic morphological characters and different aphid host spectrum than *Monoctonus* and *Harkeria*. The current results, both molecular and morphological, indicate that *H. angustivalva* should be placed in *Monoctonus*. *Monoctonus*, comprising most species of Monoctonina, can be roughly separated into three clades based on analyzed molecular markers, although morphological differences do not follow this separation in all cases. Most examined species show a high level of intraspecific diversity based on the *COI* gene.

A new species (*M. indiscretus* sp. n.) is described based on molecular data, but it is currently almost indistinguishable morphologically from related species. Three potentially new species are reported, and detailed descriptions can be made when female specimens become available. Based on morphological and molecular data, *M. ligustri* and *M. mali* are given the status of junior synonyms of *M. cerasi*, despite differences in their biology.

## Figures and Tables

**Table 1:** Genetic distances between Monoctonina species based on *COI* sequences.

Species	*M. ligustri*	*M. mali*	*M. washingtonensis*	*M. crepidis*	*M. nervosus*	*M. paulensis*	*M. leclanti*	*M. allisoni*	*M. caricis*	*M. brachyradius*	*M. inexpectatus*	*M. canadenss*	*M. luteus*	*M. parvipalpus*	*M. indiscretus*	*Monoctonus* sp.n.3	*Monoctonus* sp.n.2	*Monoctonus* sp.n.1	*H. angustivalva*	*F. pseudoplatani*	*M. vesicarii*	*M. japonica*
*M. ligustri*																						
*M. mali*	0.004																					
*M. washingtonensis*	0.163	0.165																				
*M. crepidis*	0.124	0.127	0.151																			
*M. nervosus*	0.149	0.151	0.127	0.151																		
*M. paulensis*	0.150	0.149	0.114	0.142	0.027																	
*M. leclanti*	0.181	0.182	0.193	0.191	0.198	0.199																
*M. allisoni*	0.175	0.177	0.198	0.173	0.195	0.199	0.143															
*M. caricis*	0.152	0.152	0.069	0.153	0.128	0.117	0.214	0.202														
*M. brachyradius*	0.138	0.145	0.112	0.122	0.094	0.095	0.202	0.191	0.105													
*M. inexpectatus*	0.155	0.158	0.133	0.146	0.070	0.065	0.200	0.214	0.122	0.094												
*M. canadensis*	0.142	0.143	0.136	0.138	0.131	0.137	0.181	0.184	0.134	0.149	0.157											
*M. luteus*	0.178	0.176	0.206	0.181	0.201	0.199	0.143	0.063	0.213	0.201	0.210	0.188										
*M. parvipalpus*	0.194	0.191	0.208	0.177	0.218	0.209	0.156	0.058	0.220	0.205	0.218	0.197	0.062									
*M. indiscretus*	0.159	0.164	0.126	0.156	0.131	0.137	0.216	0.209	0.134	0.123	0.138	0.140	0.221	0.219								
*Monoctonus* sp.n.3	0.150	0.151	0.069	0.153	0.115	0.099	0.214	0.198	0.046	0.097	0.110	0.140	0.204	0.209	0.129							
*Monoctonus* sp.n.2	0.152	0.159	0.124	0.152	0.117	0.120	0.201	0.184	0.121	0.105	0.123	0.167	0.205	0.201	0.129	0.112						
*Monoctonus* sp.n.1	0.186	0.186	0.199	0.187	0.206	0.203	0.141	0.035	0.199	0.191	0.206	0.196	0.069	0.066	0.213	0.197	0.185					
*H. angustivalva*	0.144	0.146	0.189	0.146	0.186	0.183	0.159	0.153	0.181	0.192	0.185	0.153	0.173	0.183	0.165	0.181	0.183	0.165				
*F. pseudoplatani*	0.161	0.168	0.152	0.155	0.150	0.157	0.206	0.214	0.159	0.156	0.171	0.130	0.221	0.224	0.156	0.146	0.165	0.217	0.180			
*M. vesicarii*	0.158	0.159	0.167	0.167	0.166	0.169	0.200	0.196	0.161	0.173	0.182	0.167	0.201	0.207	0.205	0.173	0.175	0.209	0.189	0.160		
*M. japonica*	0.155	0.164	0.178	0.167	0.167	0.174	0.205	0.207	0.171	0.164	0.183	0.156	0.214	0.232	0.191	0.183	0.184	0.225	0.176	0.158	0.045	
*M. pistaciaecola*	0.194	0.196	0.177	0.179	0.168	0.169	0.202	0.201	0.179	0.178	0.193	0.194	0.219	0.223	0.201	0.174	0.190	0.201	0.192	0.193	0.132	0.127

**Table 2:** Genetic distances between genera of Monoctonina based on *COI* sequences (below diagonal) and *28S rDNA* sequences (above diagonal).

	*Monoctonus*	*Harkeria*	*Monoctonia*	*Falciconus*
*Monoctonus*		0.043	0.180	0.156
*Harkeria*	0.176		0.163	0.141
*Monoctonia*	0.175	0.187		0.165
*Falciconus*	0.159	0.180	0.174	

**Table 3:** Genetic distances between species of Monoctonina based on *28S rDNA*.

Species	*M. ligustri*	*M. mali*	*M. crepidis*	*M. leclanti*	*M. nervosus*	*M. paulensis*	*H. angustivalva*	*F. pseudoplatani*	*M. vesicarii*	*M. japonica*
*M. ligustri*										
*M. mali*	0.000									
*M. crepidis*	0.032	0.032								
*M. leclanti*	0.036	0.036	0.040							
*M. nervosus*	0.026	0.026	0.036	0.041						
*M. paulensis*	0.044	0.044	0.048	0.049	0.004					
*H. angustivalva*	0.041	0.041	0.044	0.014	0.048	0.057				
*F. pseudoplatani*	0.149	0.149	0.154	0.143	0.165	0.200	0.141			
*M. vesicarii*	0.158	0.158	0.155	0.150	0.171	0.192	0.143	0.150		
*M. japonica*	0.164	0.164	0.160	0.153	0.168	0.200	0.153	0.154	0.003	
*M. pistaciaecola*	0.199	0.199	0.196	0.192	0.211	0.197	0.178	0.178	0.022	0.019

## Supplementary Materials

The following are available online at https://www.mdpi.com/2075-4450/11/3/160/s1, Table S1: List of samples used in the analyses.

## Appendix A

The following material represents potentially new species of Monoctonina, based on genetic distances of *COI* gene sequences. However, since only male specimens are available for analysis, we refrain from describing new species at this time, and give relevant morphological measurements which could facilitate future formal descriptions when female specimens are found.

*Monoctonus sp. n. 1*

Material examined: Canada, Alberta, Jasper National Park, 27 VI 2012, 1 male. BOLD specimen ID: CNJAC589-12.

Male

*Head*. Eyes oval, medium sized, sparsely setose. Malar space equal to approx. 0.2 of longitudinal eye diameter. Tentorial index approx. 0.4. Clypeus oval with 9 setae. Maxillary palps with four palpomeres, labial palps with three palpomeres. Antenna with 18 antennomeres, filiform, setae on flagellomeres semi-erect, subequal to half of segment diameter. F1 approx. 3.7 times as long as wide, with 3 longitudinal placodes. F2 approx. 2.7 times as long as wide, with 3–5 longitudinal placodes. F3, F4 and F5 with 4, 4–6, and 4–5 longitudinal placodes, respectively. F1 1.2 times longer than F2.

*Mesosoma*. Mesoscutum without notaulices, with sparsely setose dorsal surface. Head width/mesoscutum width ratio approx. 1.3. Propodeum areolated, with narrow central pentagonal areola and irregular oblique carinae.

*Fore wing*. Wing length approx. 2 mm, width approx. 0.8 mm. Pterostigma narrow, 6 times as long as wide and 4.1 times as long as distal abscissa of R1. Vein m-cu visible and colorless in first half, vein 2RS not visible. Veins r and 3RS distinct.

*Metasoma*. Damaged.

*Color*. Head and antenna brown, mouthparts light brown. Mesonotum, propodeum and legs brown. Wings hyaline with brown venation. Petiole light brown, rest of metasoma brown. Body length 2.3 mm.

Host: unknown.

*Distribution*: Western Canada, USA (Alaska).

*Monoctonus sp. n. 2*

Material examined: Canada, Alberta, Banff National Park, Baker Creek picnic area, 20 VI 2012, 1 male. BOLD specimen ID: SSBAB025-12.

Male

*Head*. Eyes oval, medium sized, sparsely setose. Malar space equal to approx. 0.4 of longitudinal eye diameter. Tentorial index approx. 0.5. Clypeus oval with 7 setae. Maxillary palps with three palpomeres, labial palps with two palpomeres. Antenna with 16 antennomeres, filiform, setae on flagellomeres semi-erect, subequal to half of segment diameter. F1 approx. 1.6 times as long as wide, with 6 longitudinal placodes. F2 approx. 1.6 times as long as wide, with 5–6 longitudinal placodes. F3, F4, and F5 with 5–6, 6–7, and 5–6 longitudinal placodes, respectively. F1 length equal to F2.

*Mesosoma*. Mesoscutum without notaulices, with smooth dorsal surface. Head width/mesoscutum width ratio approx. 1.2. Propodeum areolated, with narrow central pentagonal areola and irregular oblique and postmedian carinae.

*Fore wing*. Wing length approx. 1.9 mm, width approx. 0.8 mm. Pterostigma narrow, 6.8 times as long as wide and approx. 3 times as long as distal abscissa of R1. Veins m-cu and 2RS not visible. Veins r and 3RS distinct.

*Metasoma*. Petiole 1.4 times as long as wide at spiracles. Spiracular tubercles prominent. Dorsal disc of petiole moderately rugose in basal third, with 6–7 long setae on the sides.

*Colour*. Head, antenna and mouthparts brown. Mesonotum, propodeum and legs brown. Wings hyaline with brown venation. Petiole light brown, rest of metasoma brown. Body length 2 mm.

*Host*: unknown.

*Distribution*: Western Canada.

*Monoctonus sp. n. 3*

Material examined: Canada, Yukon Territory, Kluane National Park and Reserve, 15 VII 2014, 1 male. BOLD specimen ID: SSKUB5220-15; 24 VII 2014, 1 male. BOLD specimen ID: SSKUB1168-15.

Male

*Head*. Eyes oval, medium sized, sparsely setose. Malar space equal to approx. 0.2 of longitudinal eye diameter. Tentorial index approx. 0.4. Clypeus oval with 12 setae. Maxillary palps with four palpomeres, labial palps with two palpomeres. Antenna with 17–18 antennomeres, filiform, setae on flagellomeres semi-erect, subequal to half of segment diameter. F1 approx. 3.1 times as long as wide, with 6–7 longitudinal placodes. F2 approx. 2.7 times as long as wide, with 6–7 longitudinal placodes. F3, F4 and F5 with 7–8, 7–8, and 5–6 longitudinal placodes, respectively. F1 length equal to F2.

*Mesosoma*. Mesoscutum without notaulices, with smooth dorsal surface. Head width/mesoscutum width ratio approx. 1. Propodeum areolated, with narrow central pentagonal areola.

*Fore wing*. Wing length approx. 2 mm, width approx. 0.8 mm. Pterostigma narrow, 7.3 times as long as wide and 2.8 times as long as distal abscissa of R1. Vein m-cu visible and colorless, vein 2RS visible in first half. Veins r and 3RS distinct.

*Metasoma*. Petiole approx. 2.1 times as long as wide at spiracles. Spiracular tubercles prominent. Dorsal disc of petiole moderately rugose in middle third, with 5–6 long setae on the sides.

*Colour*. Head and antenna brown, mouthparts light brown. Mesonotum brown, propodeum and legs light brown. Wings hyaline with brown venation. Petiole light brown, rest of metasoma brown. Body length 2.4 mm.

*Host*: unknown.

*Distribution*: Western Canada.
